# Aneurysmal Bone Cyst after Femoral Derotational Osteotomy: A Case Report

**DOI:** 10.5704/MOJ.1903.009

**Published:** 2019-03

**Authors:** K Sahin, M Demirel, N Turgut, U Arzu, G Polat

**Affiliations:** Department of Orthopaedic and Traumatology, Istanbul Medical Faculty, Istanbul, Turkey

**Keywords:** aneurysmal bone cyst, bone cyst, bone tissue neoplasm, osteotomy

## Abstract

Aneurysmal bone cysts rather than local aggressive lesions of the bone which may arise in any part of the axial or appendicular skeleton. Although several theories are available in the literature, the pathogenesis is still conflicting. We report an exceptional case of an aneurysmal bone cyst in the distal femur of a female cerebral palsy patient who underwent bilateral distal femoral derotational osteotomy and plate-screw fixation operations when she was 11 years old. Twenty-four months after the operation, radiographs showed a cystic lesion in the distal portion of the right femur around the osteotomy site. The diagnosis of Aneurysmal Bone Cyst (ABC) was made and the lesion was treated by curettage with cement application. After 36 months of follow-up, there was no recurrence. This is the first case reported in literature which raises the possibility that an osteotomy could be a cause in the development of an aneurysmal bone cyst.

## Introduction

Aneurysmal bone cysts (ABC) are benign but not local aggressive lesions of bone which most commonly occur within the first two decades of life, with a slight female predominance. Although these lesions are seen predominantly around the knee joint, any part of the axial or appendicular skeleton may be involved^1^. The etiology of ABCs is still conflicting. Generally, ABC exists as a primary bone lesion. However, a predisposing lesion is present in 30% of the cases; a finding suggesting that ABCs are reactive lesions to other pathological changes, rather than a distinct *de novo* tumour. Moreover, trauma may also act as a pathogenic trigger for the development of ABCs^1,2^. The purpose of this case report is to present an exceptional case of an ABC of the distal femur occurring after a femoral derotational osteotomy.

## Case Report

An 11-year old girl was referred to our department with a ten-year history of difficulty in walking diagnosed as cerebral palsy (CP). The history obtained revealed that the patient was followed up regularly by paediatric neurologists for her CP, and she had multiple botulinum injections when she was 8 years old. The physical examination based on the walking analysis illustrated limited range of motion in addition to 55° of increased femoral anteversion in either hips. From the data of walking analysis, physical examination and radiological findings, the increased femoral anteversion and excessive soft tissue contractures due to CP were considered the primary reason for her walking difficulty. To improve her gait pattern, a combination of soft tissue procedure and distal femoral derotational osteotomy were planned.

Under general anaesthesia, the tendons of adductor longus, iliopsoas, semitendinosus, semimembranosus, gracilis, and gastrocnemius were released from the musculotendinous junction bilaterally. Bilateral distal femoral derotational osteotomy was then performed just above the metaphysis level with a lateral approach, and osteosynthesis was obtained by dynamic compression plate and screw fixation. Bilateral long leg casts were applied postoperatively to maintain osteosynthesis. The patient was discharged on the third post-operative day, casts were removed one month later and routine rehabilitative physiotherapy was instituted.

**Fig. 1:**
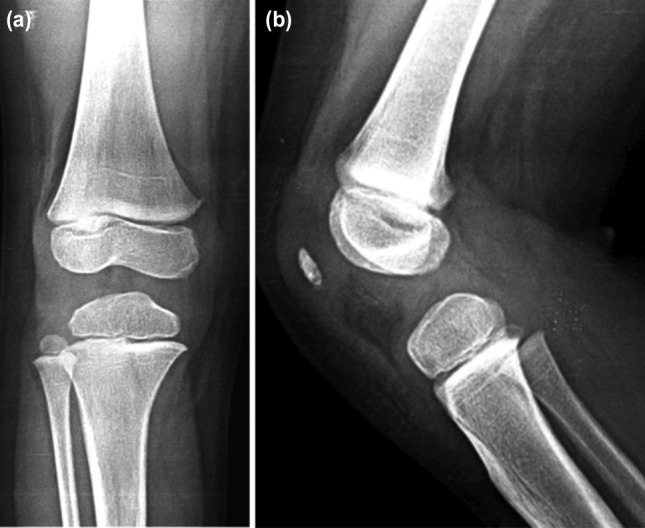
(a) Pre-operative anteroposterior and (b) lateral radiographs of the right knee before femoral derotational osteotomy surgery.

**Fig. 2:**
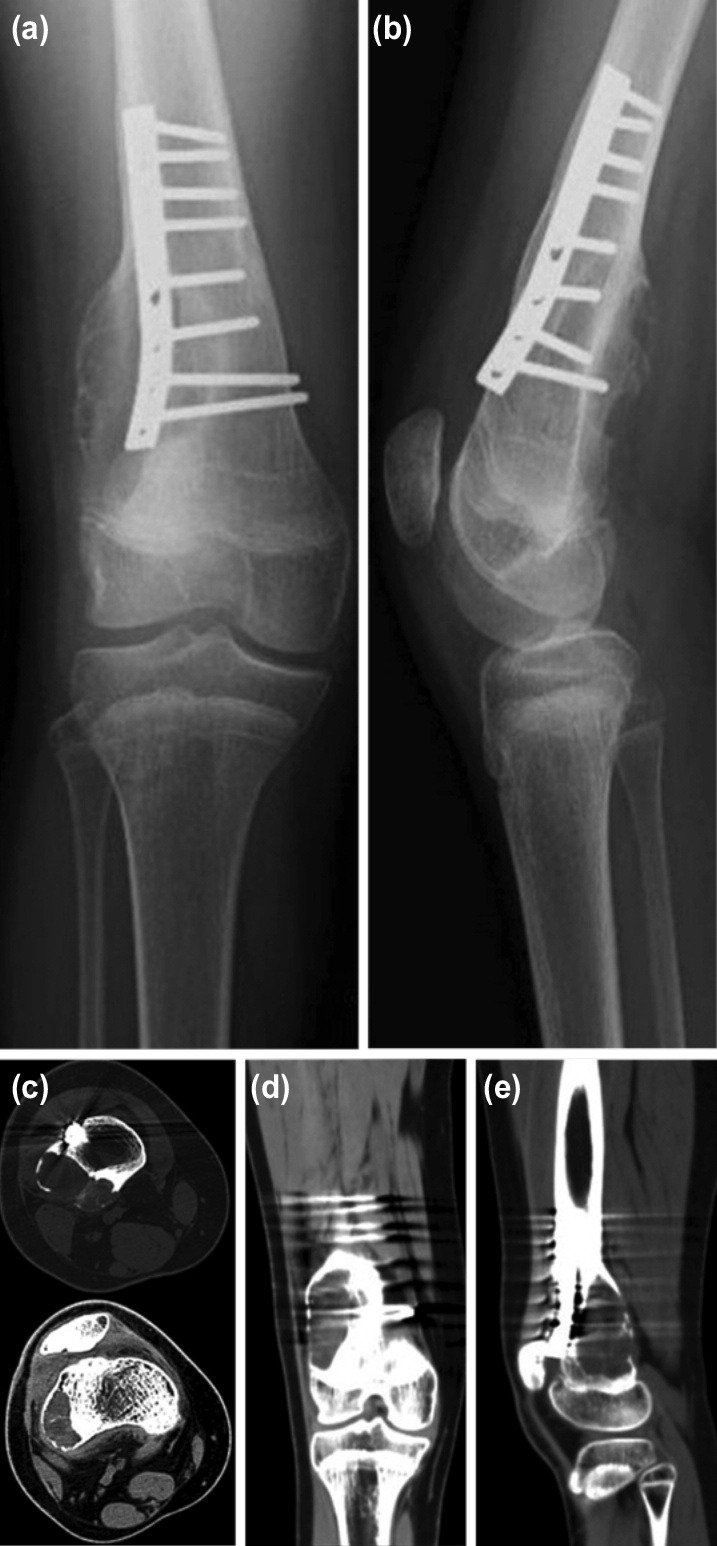
(a) Anteroposterior and (b) lateral radiographs of the right knee at the 2nd year follow-up after femoral derotational osteotomy. Radiographs show a large bone cyst on the distal lateral portion of the right femur. (c) Axial, (d) coronal and (e) sagittal computed tomography scan images of the right knee showing location and expansion of the cyst.

At one-year routine follow-up, there was complete union of the osteotomy. Although pre-operative radiographs of the right knee showed no evidence of any cystic or tumoral lesions around the distal femur (Fig. 1), the second-year follow-up radiographs revealed an asymptomatic large bone cyst in the distal part of the right femur. A computed tomography (CT) scan identified the lesion as a large cystic lesion located just above the lateral femoral condyle expanding from metaphysis to the femoral diaphysis involving the osteotomy site with cortical thinning (Fig. 2). The initial diagnosis was an aneurysmal bone cyst. To verify the diagnosis, an incisional biopsy was performed under general anaesthesia. Cortical bone was partially destroyed by the lesion and the cyst was filled with hemorrhagic material. Histopathological analysis confirmed the lesion as an ABC.

**Fig. 3:**
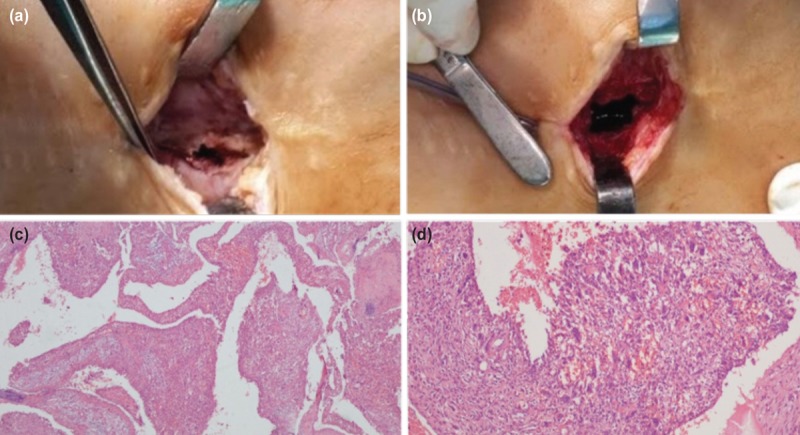
(a) Intra-operative image of the cystic lesion before curettage with visible cortical destruction of the lateral femoral cortex. (b) Image of the cyst after curettage. (c) Histopathologic view at x40 and (d) x100 magnification with hematoxylin-eosin staining showing large irregular cystic spaces, osteoclast-like multinucleated giant cells and bone tissue.

**Fig. 4:**
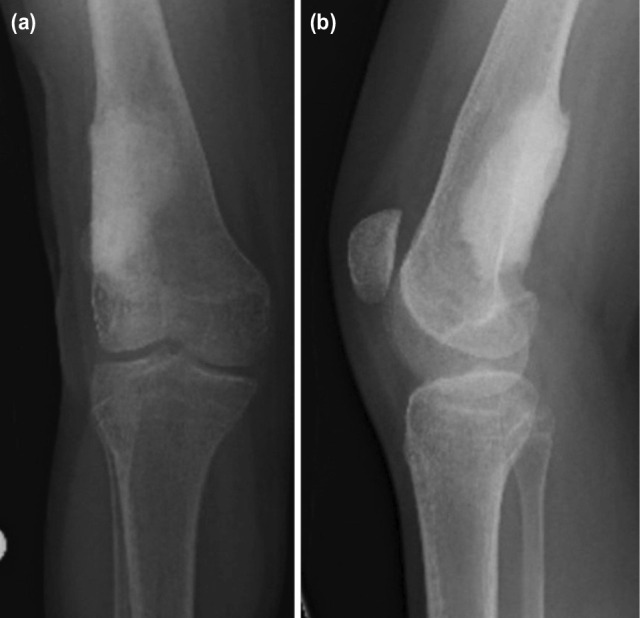
(a) Anteroposterior and (b) lateral radiographs of the right knee at the 3rd year follow-up after plate removal, curettage and cement application. Radiographs show no evidence of recurrence.

In consideration of the possible progression of the lesion and the risk of fracture associated with ABC, an operation for plate removal, curettage, and cementing procedure was performed under general anaesthesia. Histopathological examination of the curettage specimen obtained during surgery, reported as containing irregular blood-filled chambers with islands of bone and fibrous tissue, re-confirmed the diagnosis of ABC (Fig. 3). No recurrence was encountered 36 months after the curettage and cementation surgery (Fig. 4).

## Discussion

The most significant finding in the present study was that the previous osteotomy could be an underlying cause in the development of an aneurysmal bone cyst and this case is being presented as the first such case in the literature.

In previous studies, available data regarding the pathogenesis of ABC remain controversial. Numerous theories have been proposed to explain its pathogenesis, ranging from post-traumatic, reactive vascular changes to genetically predisposed bone tumors. Traditionally, ABCs are thought to arise from local circulatory disturbances leading to increased venous pressure and production of local hemorrhage^2^. However, current data suggests that two thirds of all ABCs are primary bone lesions with a neoplastic nature^3^. Furthermore, some studies suggested that ABCs may occur as a result of a hemorrhage or degeneration in patients with other bone lesions such as giant cell tumor, chondroblastoma, osteoblastoma, non-ossifying fibroma^1,2^. Additionally, it has been claimed that there may be a role of trauma as a trigger in the pathogenesis of ABC^2^. But, a limited number of studies have addressed such cases and the role of trauma in the pathogenesis is still poorly understood.

Traditional and standard treatment of ABCs include curettage of the lesion and reconstruction of the defect area with bone graft^3^. However, complications related to open surgery and high recurrence rates after curettage have resulted in the evolution of less invasive techniques and adjuvant modalities. Common adjuvant agents used in order to minimise the risk of recurrence are bone cement, high-speed burr, cryotherapy, phenol and argon beam^3^. Recently, less invasive surgical techniques including curopsy, arterial embolisation, radiotherapy, sclerotherapy and novel medical treatments such as denosumab and bisphosphonates have been investigated in order to avoid complications related to aggressive surgical procedures^3^. However, there is a lack of high-evidence study in the literature regarding the efficacy of these emerging modalities and their usage depends on the clinician’s preference. In our case, the patient was successfully treated with curettage of the lesion and application of cement without any complication or recurrence.

Trauma has been reported to be a possible etiological factor of ABCs due to the reactive vascular changes and the remodeling process of the traumatised tissues^2^. Even though the knowledge is limited to a few case reports, surgical trauma also might play a trigger role for ABC formation. Dagher *et al* reported a case of an ABC which appeared five years after repairing an acute anterior cruciate ligament rupture in the distal femoral metaphysis. The patient was treated with curettage and bone grafting. No recurrence was seen after ten months of follow-up^4^. Even though ABC was seen after surgery in this reported case, it is not possible to claim a certain causative relation between ABC formation and surgical trauma since there is a prior trauma which might also have played a role. Yamamoto *et al* reported another case of an ABC in the distal humerus which occurred after resection of an intra-articular nodular fasciitis of the elbow. The authors claimed that ABC and nodular fasciitis are characterised by similar genes and that ABC occurred predominantly secondary to nodular fasciitis. However, they admitted that surgical trauma cannot be excluded in the pathogenesis of this case^5^. In our case, surgical trauma was the sole possible etiologic factor for the formation of the ABC.

An argument can be made that the present study, which addresses the issue of ABC in the distal femur after femoral derotational osteotomy, makes an important contribution to the existing literature; because, to our knowledge, osteotomy is not yet cited as a cause of ABC in the current literature. We treated this patient successfully with curettage and cement application. After 36 months follow-up we did not see any recurrence of the ABC.

ABCs are mostly seen as primary bone lesions, however, orthopaedic surgeons should keep in mind that postsurgical trauma like osteotomy may be an etiologic factor in the development of ABCs, as in this report of our case.
